# Relative Importance of the Arcuate and Anteroventral Periventricular Kisspeptin Neurons in Control of Puberty and Reproductive Function in Female Rats

**DOI:** 10.1210/en.2014-1655

**Published:** 2015-04-15

**Authors:** M. H. Hu, X. F. Li, B. McCausland, S. Y. Li, R. Gresham, J. S. Kinsey-Jones, J. V. Gardiner, A. H. Sam, S. R. Bloom, L. Poston, S. L. Lightman, K. G. Murphy, K. T. O'Byrne

**Affiliations:** Division of Women's Health (M.H.H., X.F.L., B.M., S.Y.L., R.G., L.P., K.T.O.), Faculty of Life Sciences and Medicine, King's College London, Guy's Campus, London SE1 1UL, United Kingdom; Section of Investigative Medicine (J.S.K.-J., J.V.G., A.H.S., S.R.B., K.G.M.), Division of Diabetes, Endocrinology, and Metabolism, Imperial College London, London W12 0NN, United Kingdom; and Henry Wellcome Laboratory for Integrative Neuroscience and Endocrinology (S.L.L.), University of Bristol, Bristol BS13NY, United Kingdom

## Abstract

Kisspeptin plays a critical role in pubertal timing and reproductive function. In rodents, kisspeptin perikarya within the hypothalamic arcuate (ARC) and anteroventral periventricular (AVPV) nuclei are thought to be involved in LH pulse and surge generation, respectively. Using bilateral microinjections of recombinant adeno-associated virus encoding kisspeptin antisense into the ARC or AVPV of female rats at postnatal day 10, we investigated the relative importance of these two kisspeptin populations in the control of pubertal timing, estrous cyclicity, and LH surge and pulse generation. A 37% knockdown of kisspeptin in the AVPV resulted in a significant delay in vaginal opening and first vaginal estrous, abnormal estrous cyclicity, and reduction in the occurrence of spontaneous LH surges, although these retained normal amplitude. This AVPV knockdown had no effect on LH pulse frequency, measured after ovariectomy. A 32% reduction of kisspeptin in the ARC had no effect on the onset of puberty but resulted in abnormal estrous cyclicity and decreased LH pulse frequency. Additionally, the knockdown of kisspeptin in the ARC decreased the amplitude but not the incidence of LH surges. These results might suggest that the role of AVPV kisspeptin in the control of pubertal timing is particularly sensitive to perturbation. In accordance with our previous studies, ARC kisspeptin signaling was critical for normal pulsatile LH secretion in female rats. Despite the widely reported role of AVPV kisspeptin neurons in LH surge generation, this study suggests that both AVPV and ARC populations are essential for normal LH surges and estrous cyclicity.

Kisspeptin (Kiss1) is essential for sexual maturation because inactivating mutations in *Kiss1* (1) or its receptor (*Kiss1r*) (2, 3) result in a lack of puberty and hypogonadotropic hypogonadism, and activating mutations cause precocious puberty (4, 5). Kisspeptin is the most potent upstream stimulator of GnRH across many species (6–10) and is thought to act upon GnRH neurons directly; most GnRH neurons express *Kiss1r* mRNA (11, 12) and Kiss1 induces GnRH neuron c-Fos expression in rats (10). Signaling through the GnRH neuron Kiss1r has been shown to be essential for puberty onset and fertility (13, 14).

In rodents kisspeptin perikarya are located in the arcuate (ARC) and anteroventral periventricular (AVPV) hypothalamic nuclei, regions thought to have distinct roles in kisspeptin-GnRH function. Unlike GnRH neurons, both ARC and AVPV kisspeptin neurons express estrogen receptor (ER)-α, acting as relay afferents for estrogenic feedback upon the hypothalamus, positive feedback via the AVPV and negative via the ARC (15, 16). AVPV neurons project onto and directly stimulate preoptic area (POA) GnRH neurons (11). In rodents, the AVPV has a well-established role in LH surge generation; the POA infusion of a kisspeptin-specific antibody blocks the LH surge and represses estrous cyclicity, and kisspeptin infusion can induce a surge equivalent of LH (17). Recently kisspeptin has been shown to successfully induce an LH surge and egg maturation in humans, with the potential for use in in vitro fertilization techniques (18).

ARC kisspeptin neurons are implicated in GnRH pulse generation and are unique in that they coexpress neurokinin B (NKB) and the opioid dynorphin A (Dyn) and are thus denoted kisspeptin-NKB-Dyn (KNDy) neurons (19). Oscillations of NKB and Dyn are thought to respectively stimulate and inhibit KNDy neurons, driving the pulsed release of kisspeptin, which acts in turn to drive pulses of GnRH released at the median eminence (19). Axo-axonal interactions may occur between GnRH and kisspeptin neurons at the median eminence (20, 21), and kisspeptin and GnRH release in this region have high temporal concordance in rhesus monkeys (22). Kisspeptin administration has been shown to increase LH pulse frequency in humans (23, 24), and infusion of a kisspeptin antagonist into the median eminence suppresses GnRH pulses in female rhesus monkeys (25). Intra-ARC administration of a kisspeptin antagonist dose dependently reduces LH pulse frequency in rats (26). We have recently shown that a 27% reduction in ARC kisspeptin, using a similar protocol of stereotactic injection of antisense kisspeptin in adult Wistar rats, was sufficient to, albeit modestly, significantly decrease LH pulse frequency without affecting pulse amplitude (27). However, humans with severe inactivating mutations in *KISS1* or *KISS1R* retain very low amplitude, although with normal frequency LH pulses (28, 29). KISS1 may therefore amplify GnRH action, modulating GnRH amplitude.

GnRH pulse amplitude and frequency increase over puberty, stimulating gonadal maturation (30, 31). Kisspeptin antagonists can delay (32) and agonists advance (33) puberty onset in rats. Over the pubertal transition, LH minisurges occur in the afternoon, at approximately the time of the adult surge, increasing in amplitude and frequency until the first mature LH surge proper (34). Estrogen-dependent maturation of AVPV *Kiss1* gene expression (35), AVPV *Kiss1* mRNA and immunoreactivity (7, 36, 37), AVPV *Kiss1* promoter histone 3 acetylation, recruiting ERα (38), GnRH neuron kisspeptin appositions (39), *Kiss1r* expression (12), and GnRH neuron sensitivity to kisspeptin (36) occur over puberty.

ARC *Kiss1* mRNA increases over puberty in the rat and mouse to a much lesser degree than AVPV *Kiss1* mRNA (35, 40, 41). However, increasing pulse generator frequency is a key pubertal event in the rat (30, 40). Although the roles of ARC and AVPV nuclei in LH surge and pulse generation have been explored, whether it is ARC or AVPV kisspeptin, which is essential for puberty onset is unknown; their distinct roles in subsequent estrous cyclicity have also not been directly investigated. So far, congenital cell ablation and gene knockout methods have been used to study the necessity of kisspeptin in puberty. Whole-body kisspeptin congenital ablation, kisspeptin gene knockout, and specific GnRH neuron Kiss1r deletion methods have been used previously, with results respectively interpreted as revealing functional compensation, redundant overproduction of kisspeptin, or the necessity of Kiss1r on the GnRH neuron for puberty, with some conflicting results.

Large-scale gross knockdown methods do not allow discernment of the regional actions of kisspeptin. In this study we used microinjections of antisense kisspeptin cDNA in a recombinant AAV-associated viral construct (rAAV-kisspeptin-AS) bilaterally into either ARC or AVPV nuclei of female Sprague Dawley rats on postnatal day (pnd) 10, using rAAV-encoding enhanced green fluorescent protein (rAAV-EGFP) as a control. Unlike global knockdown models, local injections allow discernment of the regional actions of Kiss1 neurons; some physiological function of kisspeptin is retained which, in combination with injecting the rats at this age, minimizes potential confounding by functional developmental compensation. We were therefore able to investigate the differential role of ARC and AVPV kisspeptin signaling in pubertal timing, estrous cyclicity, and LH surge and pulse generation.

## Materials and Methods

Female Sprague Dawley rats, obtained from Charles River, fed standard laboratory chow, and watered ad libitum, were bred and housed with their litters under controlled conditions (temperature, 21–23ºC, 12-hour light, 12-hour dark cycles with lights on at 7:00 am) until pnd 21 when the female pups were weaned and housed in groups of three to four per cage. The body weight of all female pups was measured every 3 days from weaning. All animal procedures were in accordance with the British Home Office Animals Scientific Procedures Act 1986.

### Antisense kisspeptin construct

RT-PCR isolated full-length *Kiss1* cDNA was cloned into pTR-CGW vector (from Dr J. Verhaagen, Netherlands Institute for Neuroscience, Amsterdam, The Netherlands) with the ampicillin resistance gene, inverted terminal repeats, human immediate early cytomegalovirus promoter and wood chuck posttranscriptional regulatory element. The cytomegalovirus promoter was reverse orientation, producing antisense cRNA. We have previously shown the validity of this construct in inhibiting kisspeptin production (27). Both rAAV-kisspeptin-AS and rAAV-EGFP were prepared using the adenovirus-free method described previously (42) and rAAV was produced using the two-plasmid system (plasmid pDG from Dr J. A. Kleinschmidt, German Cancer Research Center, Heidelberg, Germany). The kisspeptin-AS corresponds to nucleotides 1–393 of sequence NM-181692. Dot blot assays were used to establish physical particle titer, and infectious center assays were used to determine the infectious particle titer.

### Intranuclear injection

Surgery was performed at pnd 10 to allow 3 weeks for maximum expression of kisspeptin-AS to be achieved before puberty onset (27, 42, 43). Surgical procedures were performed under aseptic conditions, using general anesthesia (ketamine hydrochloride USP; 100 mg/mL; Phizer Ltd) and Rompun (10 mg/kg; Bayer AG) via ip injection. The animal was secured in a David Kopf stereotaxic frame. Using a 2-μL Hamilton microsyringe (Sigma-Aldrich Ltd), with a glass needle connected to its tip, 1 μL of rAAV-kisspeptin-AS or rAAV-EGFP containing 5 × 10^12^ genome particles were infused into either the ARC or the AVPV nucleus over 10 minutes, via a burr hole (FFXC7HP burr; Wright Dental Group Ltd) drilled into the skull overlying the ARC or AVPV nucleus. The needle was left in place for 5 minutes and then removed slowly over 1 minute and the procedure repeated for the contralateral nucleus. The respective coordinates for the ARC and AVPV (0.38 mm lateral, 2.60 mm posterior to bregma, 7.30 mm below the surface of the dura, and 0.38 mm lateral, 0.18 mm anterior to bregma, 6.10 mm below the surface of the dura) were used after careful verification in preliminary experiments and dye injection sites in brain sections of pnd 10 rats, targeting the ARC and AVPV using initial coordinates 0.50 mm lateral, 3.30 mm posterior to bregma, 10.50 mm below the surface of the dura, and 0.20 mm lateral, 0.26 mm posterior to bregma, 8.60 mm below the surface of the dura, respectively, as per the adult rat atlas (44) to establish coordinates for the smaller animals.

At 3 weeks after injection of rAAV-EGFP into the ARC or AVPV, a subset of rats (n = 5 per group) was killed, brains removed, and stored at −80ºC. Forty-micrometer coronal sections were cut and mounted on polylysine slides. The brain sections were rehydrated, coverslipped, and viewed by direct fluorescence using a microscope (Nikon A1R Si Confocal; Nikon).

### Puberty onset and estrous cycles

Animals were monitored for vaginal opening and first vaginal estrus (markers of puberty onset) between 9:00 and 10:00 am daily from pnd 28. Once vaginal opening occurred, vaginal smears were taken daily for 2–3 consecutive weeks to detect the stage of the estrous cycles. Normal estrous cyclicity was defined as having at least two consecutive normal cycles, which lasted for 4–5 days with 1–2 days of estrus. Cycle length, with three stages observed in the correct order, was determined by the number of days between each occurrence of estrus.

### Blood sampling to detect LH and estradiol levels

Approximately 7 days after puberty, the rats were implanted with two indwelling cardiac catheters via the jugular veins to enable blood sampling for LH and estradiol measurement. The catheters were exteriorized at the back of the head and secured to a cranial attachment (40); the rats were fitted with a 30-cm-long metal spring tether (Instec Laboratories Inc), the distal end of which was attached to a fluid swivel (Instec Laboratories), allowing the rat free movement. After iv catheterization, rats were housed individually and blood sampling commenced about 3 days later.

### LH surge

For spontaneous LH surge detection approximately 10 days after puberty onset, once confirmed by vaginal smear to be in the proestrous cycle stage, the rats were connected by one of the two cardiac catheters to a computerized automatic blood sampling system. Half-hourly blood sampling began at 11:30 am for 8 hours. At every 25-μL blood withdrawal, 25 μL of heparinized saline (50 U/mL heparin sodium normal saline (Wockhardt) was infused into the rat to keep the catheter patent and restore blood volume. Manual blood samples (250 μL) were collected via the second catheter at 12:00 pm for estradiol measurement. Blood samples were frozen at −20ºC for later LH and estradiol assay.

### LH pulses

After the blood sample collection for the detection of the spontaneous LH surge, rats were ovariectomized (OVX) (45) to facilitate LH pulse detection. After a 10-day recovery period, they underwent automated blood sampling for LH pulse frequency, starting at 10:00 am, sampling every 5 minutes for 6 hours. The animals were then killed and tissues collected.

### Quantification of ARC and AVPV kisspeptin and ARC NKB

Animals were decapitated on pnd 50–60 and brains rapidly removed and snap frozen in isopentane and stored at −80ºC. Coronal sections (300 μm) were cut on a cryostat, and punches (1 mm diameter) of these serial sections of the ARC and AVPV were taken from bregma −1.7 to −3.9 mm and +0.2 to −0.4 mm, respectively, according to the rat brain atlas (44), after the micropunch method of Palkovits (46). The punched tissue was homogenized in an acid-ethanol extraction buffer (0.15% hydrochloric acid in 25% ethanol) and analyzed for the quantity of kisspeptin by specific kisspeptin RIA using 1.5 × 10^−5^ dilution Millipore kisspeptin rabbit polyclonal antibody (Millipore) raised to kisspeptin-10 and^125^I-labeled kisspeptin-10 (Advanced Biotechnology Centre, Imperial College London, London, United Kingdom) as described previously (27, 47) (see Table 1). All assays used 350 μL of phosphate buffer (pH 7.4) with 0.3% BSA and 0.02% Tween 20 and had 3 days of incubation. The smallest detectable limit for the assays was 1 fmol/punch. NKB was quantified in punched ARC tissue from intra-ARC rAAV-kisspeptin-AS- and control rAAV-EGFP-injected animals, using an ELISA kit following the manufacturer's instructions (Peninsula Laboratories LLC).

**Table 1.** Information About Antibodies Used

Peptide Target	Antigen Sequence	Name of Antibody	Manufacturer, Catalog Number, and/or Name of Individual Providing the Antibody	Species Raised (Monoclonal or Polyclonal)	Dilution Used
Kisspeptin 10	YNWNSFGLRY-NH2	Antikisspeptin	Millipore, AB9754	Rabbit, polyclonal	1:1.5 × 10^−5^

### RIA for LH and estradiol

A double-antibody RIA supplied by the National Institute of Diabetes and Digestive and Kidney Diseases (Bethesda, Maryland) was used to determine the LH concentration in the 25-μL whole-blood sample. Reference preparation was rat Luteinizing Hormone-Reference Preparation-3 (rLH-RP-3). The sensitivity of the assay was 0.093 ng/mL. The intraassay coefficient of variation was 5.3% and the interassay coefficient of variation was 7.5%. A double-antibody RIA (ImmuChem; MP Biomedicals) was used to estimate the estradiol content of the plasma samples (50 μL) following the manufacturer's protocol. The intraassay coefficient of variation was 10.2%. All samples were analyzed on single determinations.

### Statistical analysis

All data are presented as the mean ± SEM. The effect of rAAV-kisspeptin-AS on LH surges was determined by analyzing the area under the LH profile curve (AUC). The algorithm ULTRA was used to establish the detection of LH pulses (48). Two intraassay coefficients of variation of the assay were used as the reference threshold for the pulse detection. The effect of rAAV-kisspeptin-AS on pulsatile LH secretion was analyzed by comparing the mean LH pulse interval over the 6-hour sampling period. A Student's *t* test was used to analyze kisspeptin immunoreactivity and NKB levels. A Fisher's test was used to analyze the percentage of rats spontaneously exhibiting an LH surge in proestrus and for analysis of estrous cyclicity. All other results were analyzed by a one-way ANOVA (Systat Software). *P* < .05 was considered significant.

## Results

### Anatomical localization of enhanced green fluorescent protein (EGFP) after ARC or AVPV injection

**Figure 1.**
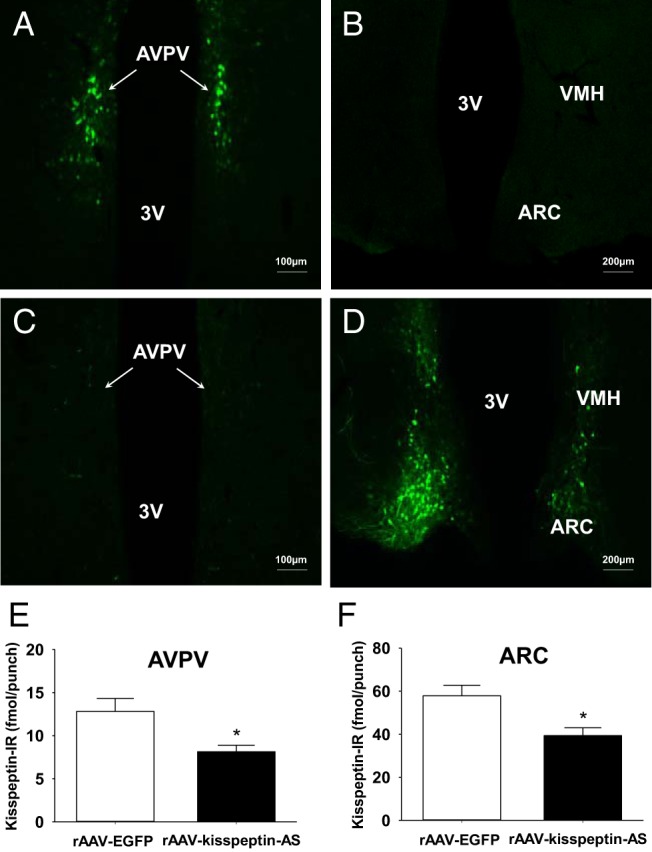
Effect of intra-ARC and intra-AVPV administration of rAAV-kisspeptin-AS or rAAV-EGFP in female rats. Representative images showing rAAV-EGFP spread in the AVPV (A) and ARC (B) after intra-AVPV administration are shown. Representative images showing rAAV-EGFP spread in the AVPV (C) and ARC (D) after intra-ARC administration are also shown. 3V, third ventricle; VMH, ventromedial hypothalamus. Scale bars represent 100 μm (A and C) and 200 μm (B and D), respectively. E and F, The effect of intra-AVPV or intra-ARC rAAV-EGFP and rAAV-kisspeptin-AS administration (on pnd 10) on kisspeptin-IR in the AVPV and ARC, respectively. Quantification of kisspeptin-IR in the AVPV and ARC was carried out between pnd 50 and pnd 60 by a RIA (n = 7–9 per group). Data are shown as means ± SEM. *, *P* < .05.

After intra-ARC or intra-AVPV rAAV-EGFP injection, EGFP-containing cell bodies and axons were observed at high density within the ARC and AVPV, respectively, without spread to surrounding hypothalamic nuclei (Figure 1, A–D). Some slight expression of EGFP was also observed in the injection tracts. Critically, for the intra-ARC- or intra-AVPV rAAV-EGFP-injected animals, no EGFP-containing neurones were observed in the corresponding AVPV or ARC, respectively (Figure 1, A–D).

### Effect of rAAV-kisspeptin-AS on ARC and AVPV kisspeptin immunoreactivity (IR) and ARC levels of NKB

Intra-ARC and intra-AVPV rAAV-kisspeptin-AS injections both resulted in significant reductions in kisspeptin immunoreactivity (IR), validated and quantified by micropunch collection and RIA. Intra-ARC rAAV-kisspeptin-AS caused a 32.0% knockdown in ARC kisspeptin-IR vs control rAAV-EGFP rats [39.4 ± 3.7 (ARC rAAV-kisspeptin-AS; n = 7) vs 57.9 ± 4.9 (ARC rAAV-EGFP; n = 8) fmol/punch, *P* < .05, Figure 1F]. Intra-AVPV rAAV-kisspeptin-AS resulted in a 36.5% reduction in AVPV kisspeptin-IR vs rAAV-EGFP controls [8.1 ± 0.8 (rAAV-kisspeptin-AS; n = 7) vs 12.8 ± 1.5 (rAAV-EGFP; n = 9) fmol/punch, *P* < .05, Figure 1E]. As an additional control for potential cross-contamination between the injection sites in the ARC and AVPV, the effects of intra-ARC or intra-AVPV rAAV-kisspeptin-AS injections on kisspeptin-IR in the opposite nucleus to where the virus was injected were examined and shown to have no affect compared with the respective rAAV-EGFP controls (ARC content after intra-AVPV rAAV-kisspeptin-AS: 68.3 ± 8.4 fmol/punch, n = 6, *P* > .05; AVPV content after intra-ARC rAAV-kisspeptin-AS: 16.0 ± 1.2 fmol/punch, n = 6, *P* > .05). Intra-ARC administration of rAAV-kisspeptin-AS had no significant effect on ARC-NKB content compared with rAAV-EGFP control [31.3 ± 6.5 pg/punch (rAAV-EGFP) vs 27.6 ± 4.8 pg/punch (rAAV-kisspeptin-AS; n = 6–7), *P* > .05].

### Effect of rAAV-kisspeptin-AS on female rat puberty

**Figure 2.**
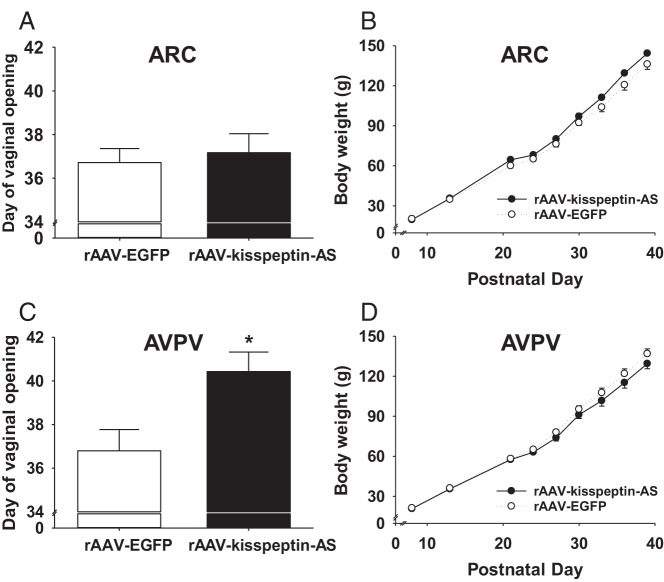
The effect of intra-ARC and intra-AVPV rAAV-kisspeptin-AS administration on day of vaginal opening and body weight in rats. A, No significant delay was observed in day of vaginal opening in intra-ARC rAAV-kisspeptin-AS vs intra-ARC rAAV-EGFP controls. B, Body weight did not vary significantly between ARC rAAV-kisspeptin-AS and ARC rAAV-EGFP. C, There was a significant delay in day of vaginal opening after intra-AVPV rAAV-Kisspeptin-AS compared with intra-AVPV rAAV-EGFP controls. D, Body weight did not vary significantly between AVPV rAAV-kisspeptin-AS and AVPV rAAV-EGFP. Results are presented as means ± SEM. *, *P* < .05 vs rAAV-EGFP controls (means ± SEM; n = 7–9 per group).

Intra-ARC rAAV-kisspeptin-AS did not significantly alter the day of vaginal opening compared with ARC rAAV-EGFP controls [37.2 ± 0.9 (ARC rAAV-kisspeptin-AS; n = 7) vs 36.7 ± 0.6 (ARC rAAV-EGFP; n = 8) postnatal days, *P* = .68, Figure 2A] or the day of first vaginal estrus [37.3 ± 0.7 (ARC rAAV-kisspeptin-AS; n = 9) vs 37.5 ± 1.0 (ARC rAAV-EGFP; n = 7) postnatal days, *P* = .86]. There was no significant difference between gross body weight gain between ARC rAAV-kisspeptin-AS rats and controls (Figure 2B).

There was a significant delay in the day of vaginal opening after intra-AVPV rAAV-kisspeptin-AS administration compared with AVPV rAAV-EGFP controls [40.2 ± 0.9 (AVPV rAAV-kisspeptin-AS; n = 7) vs 36.8 ± 1.0 (AVPV rAAV-EGFP; n = 8) postnatal days, *P* < .05, Figure 2C]. Intra-AVPV rAAV-kisspeptin-AS also caused a significant delay in day of first vaginal estrus vs rAAV-EGFP controls [41.0 ± 0.9 (AVPV rAAV-kisspeptin-AS; n = 7) vs 37.2 ± 1.0 (AVPV rAAV-EGFP; n = 9) postnatal days, *P* < .05]. Gross body weight gain did not vary significantly between AVPV rAAV-kisspeptin-AS rats and controls (Figure 2D).

### Effect of rAAV-kisspeptin-AS on ovarian cyclicity

**Figure 3.**
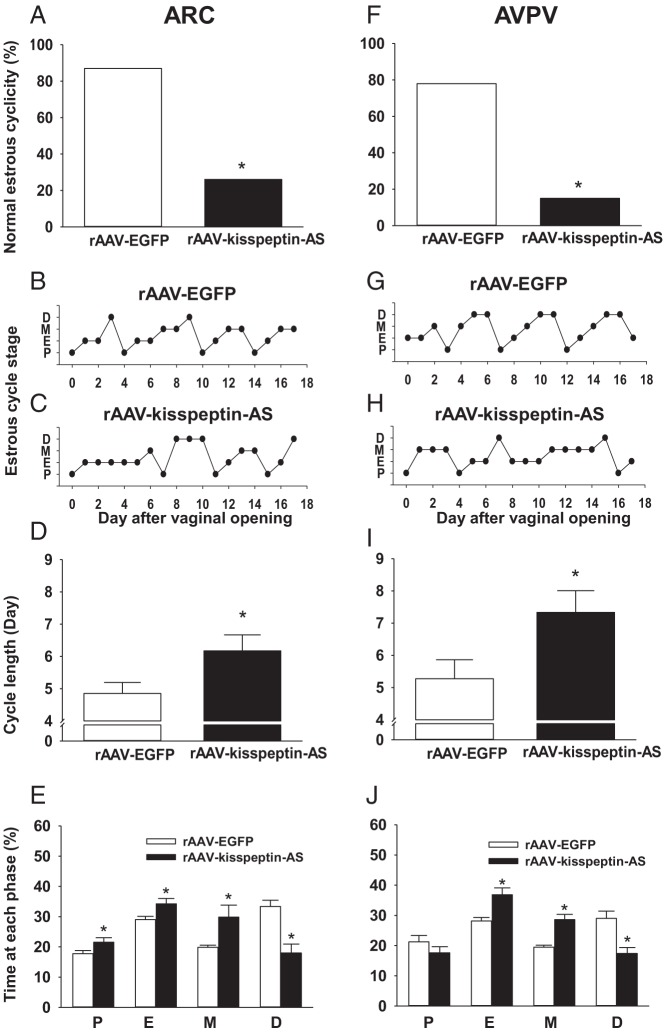
The effect of intra-ARC and intra-AVPV rAAV-kisspeptin-AS administration on estrous cyclicity in juvenile rats. Cyclicity was determined by analyzing daily vaginal cytology from day of vaginal opening to ovariectomy. A, ARC rAAV-kisspeptin-AS resulted in a significant decrease in percentage of normal estrous cyclicity vs rAAV-EGFP controls. Representative examples of estrous cyclicity are presented for ARC rAAV-EGFP (B) and ARC rAAV-kisspeptin-AS (C) rats. D, Cycle length was significantly extended after intra-ARC rAAV-kisspeptin-AS vs intra-ARC rAAV-EGFP controls. E, Time spent in proestrous, estrous, and metestrous stages was extended, whereas time spent in diestrus was decreased by ARC rAAV-kisspeptin-AS compared with controls. F, AVPV rAAV-kisspeptin-AS also resulted in a significant decrease in percentage of normal estrous cyclicity vs rAAV-EGFP controls. Representative examples of estrous cyclicity are presented for AVPV rAAV-EGFP (G) and AVPV rAAV-kisspeptin-AS (H) rats. I, Intra-AVPV rAAV-kisspeptin-AS also significantly extended the cycle length compared with intra-AVPV rAAV-EGFP. J, Intra-AVPV rAAV-kisspeptin-AS did not significantly extend the percentage of time spent in proestrus compared with AVPV rAAV-EGFP. Both estrous and metestrous cycle stages were prolonged after AVPV rAAV-kisspeptin-AS vs AVPV rAAV-EGFP. AVPV rAAV-kisspeptin-AS reduced time spent in diestrus. Results are presented as means ± SEM. *, *P* < .05 vs rAAV-EGFP controls (means ± SEM; n = 7–9 per group).

Daily vaginal cytology from puberty onset until the rats were ovariectomized revealed a significant reduction in the percentage of normal estrous cycles after both ARC and AVPV rAAV-kisspeptin-AS administration. Intra-ARC rAAV-kisspeptin-AS injected rats compared with rAAV-EGFP controls had significantly fewer normal estrous cycles [percentage of normal estrous cyclicity (28.5% [ARC rAAV-kisspeptin-AS; n = 7] vs 87.5% [ARC rAAV-EGFP; n = 8] *P* < .05, Figure 3A]. Representative examples of typical estrous cyclicity are provided (Figure 3B, ARC rAAV-EGFP, and Figure 3C, ARC rAAV-kisspeptin-AS). Cycle length was also prolonged (Figure 3D; *P* < .05) with an increase in estrus and decrease in diestrous phases evident in intra-ARC rAAV-kisspeptin-AS-treated animals (Figure 3E; *P* < .05). Normal estrous cyclicity was also significantly reduced in intra-AVPV rAAV-kisspeptin-AS rats compared with rAAV-EGFP controls during the experimental period [percentage of normal estrous cyclicity (14.2% [AVPV rAAV-kisspeptin-AS; n = 7] vs 78.0% [AVPV rAAV-EGFP; n = 9], *P* < .05, Figure 3F]. Examples of typical estrous cyclicity are provided (Figure 3G, AVPV rAAV-EGFP, and Figure 3H, AVPV rAAV-kisspeptin-AS). Intra-AVPV rAAV-kisspeptin-AS similarly significantly extended cycle length (Figure 3I; *P* < .05) with an increase in estrus and decrease in diestrous phases evident (Figure 3J; *P* < .05).

### Effect of rAAV-kisspeptin-AS on spontaneous LH surges

**Figure 4.**
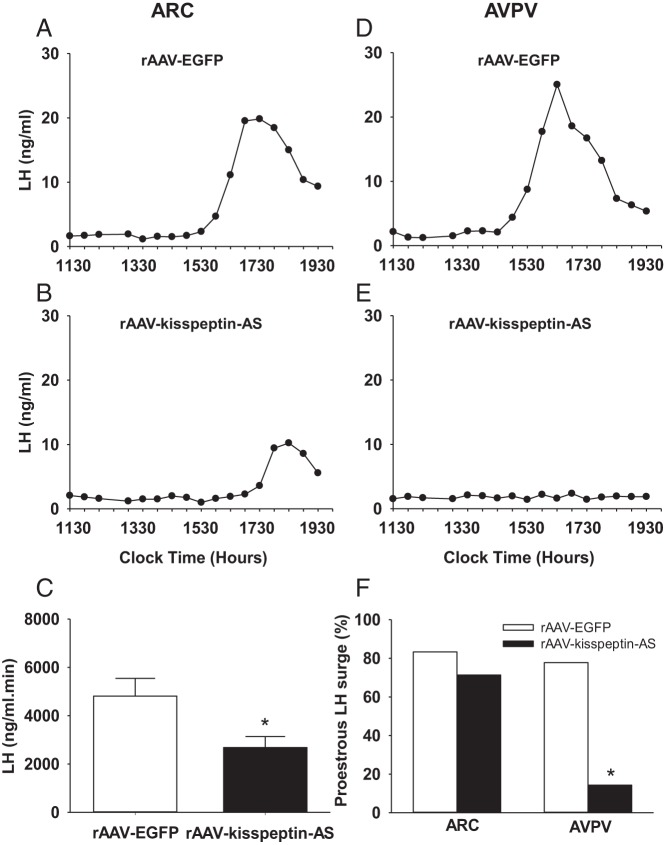
The effect of intra-ARC and intra-AVPV rAAV-kisspeptin-AS administration on LH surges in rats. Representative LH surges after intra-ARC rAAV-EGFP (A) and intra-ARC rAAV-kisspeptin-AS (B) are shown. C, AUC analysis of LH surges after intra-ARC rAAV-kisspeptin-AS vs control intra-ARC rAAV-EGFP revealed a significant decrease after ARC kisspeptin knockdown. Representative LH surge for intra-AVPV rAAV-EGFP (D) and the absence of an LH surge (E) after intra-AVPV rAAV-kisspeptin-AS administration are also shown. F, Intra-AVPV rAAV-kisspeptin-AS significantly decreased proestrous LH surge incidence vs intra-AVPV rAAV-EGFP controls; only one of seven rats exhibiting a normal LH surge occurring on proestrus. There was no significant difference in incidence of LH surges occurring on proestrus after intra-ARC rAAV-kisspeptin-AS vs intra-ARC rAAV-EGFP controls (F). Results are presented as means ± SEM. *, *P* < .05 vs rAAV-EGFP controls (means ± SEM; n = 5–9 per group).

To assess LH surge regularity, the percentage of rats exhibiting an LH surge on the day of proestrus was analyzed for each experimental group. Spontaneous LH surges were detected in most control rats on the day of proestrous; no significant reduction was found in ARC rAAV-kisspeptin-AS animals vs ARC rAAV-EGFP controls [71.4% (ARC rAAV-kisspeptin-AS; n = 7) vs 83.3% (ARC rAAV-EGFP; n = 6) *P* = .61, Figure 4F]. However, an AUC analysis showed a significant decrease in LH surge amplitude after ARC rAAV-kisspeptin-AS administration [2680.84 ± 457.09 (ARC rAAV-kisspeptin-AS; n = 5) vs 4814.47 ± 733.10 (ARC rAAV-EGFP; n = 5) ng/mL · min, *P* < .05, Figure 4C]. Representative examples of ARC rAAV-EGFP control (Figure 4A) and rAAV-kisspeptin-AS (Figure 4B) LH surges are included. Estradiol levels measured on the same proestrous day as the detection of the LH surge were significantly decreased in the ARC rAAV-kisspeptin-AS rats [23.9 ± 2.4 (ARC rAAV-kisspeptin-AS; n = 7) vs 34.1 ± 4.0 (ARC rAAV-EGFP; n = 6) pg/mL, *P* < .05].

Representative examples of AVPV rAAV-EGFP (Figure 4D) and rAAV-kisspeptin-AS (Figure 4E) proestrous LH profiles are included. For the AVPV rAAV-kisspeptin-AS group, the representative profile demonstrates an absence of an LH surge (Figure 4E) because only one of the seven rats assessed exhibited a surge on proestrus; this is reflected in the significant percentage reduction in LH surge regularity induced after the AVPV rAAV-kisspeptin-AS injection compared with the rAAV-EGFP controls [14.3% (AVPV rAAV-kisspeptin-AS; n = 7) vs 77.8% (AVPV rAAV-EGFP; n = 9) *P* < .05, Figure 4F]. There was no significant difference between ARC and AVPV control LH surge AUC analyses, which were comparable in size with the one LH surge exhibited among the AVPV rAAV-kisspeptin-AS group. Estradiol levels measured on the same proestrous day as detection of the LH surge were significantly decreased in the AVPV rAAV-kisspeptin-AS rats [20.8 ± 2.6 (AVPV rAAV-kisspeptin-AS; n = 7) vs 38.3 ± 3.5 (AVPV rAAV-EGFP; n = 9) pg/mL, *P* < .05].

### Effect of rAAV-kisspeptin-AS on pulsatile LH release in adult OVX rats

**Figure 5.**
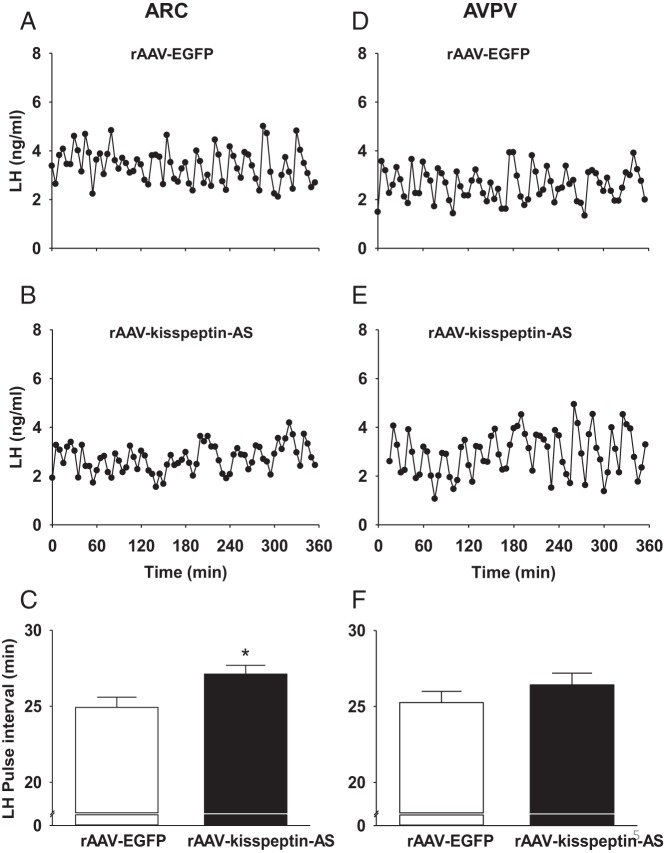
The effect of intra-ARC and intra-AVPV rAAV-kisspeptin-AS on pulsatile LH release in OVX female rats. Representative LH profiles illustrating the effect of intra-ARC rAAV-EGFP (A) and intra-ARC rAAV-kisspeptin-AS (B) are shown. C, Intra-ARC rAAV-kisspeptin-AS significantly increased LH pulse interval compared with ARC rAAV-EGFP controls. Representative LH profiles illustrating the effect of intra-AVPV rAAV-EGFP (D) and intra-AVPV rAAV-kisspeptin-AS (E) are also shown. F, LH pulse interval was not significantly altered by intra-AVPV rAAV-kisspeptin-AS compared with rAAV-EGFP controls. Results are presented as means ± SEM. *, *P* < .05 vs rAAV-EGFP controls (means ± SEM; n = 8–9 per group).

Regular LH pulses were detected in all OVX experimental groups. Representative LH profiles illustrating the effect of intra-ARC rAAV-EGFP (Figure 5A) and intra-ARC rAAV-kisspeptin-AS (Figure 5B) are included. OVX rats administered intra-ARC rAAV-kisspeptin-AS had significantly longer LH pulse intervals compared with ARC rAAV-EGFP controls [27.1 ± 0.6 (ARC rAAV-kisspeptin-AS; n = 7) vs 24.9 ± 0.5 (ARC rAAV-EGFP; n = 8) min, *P* < .05, Figure 5C]. Representative LH profiles for animals administered intra-ARC rAAV-EGFP (Figure 5D) and intra-ARC rAAV-kisspeptin-AS (Figure 5E) are included. Intra-AVPV rAAV-kisspeptin-AS animals exhibited no significant difference in LH pulse intervals vs controls [26.4 ± 0.8 (AVPV rAAV-kisspeptin-AS; n = 7) vs 25.3 ± 0.7 (AVPV rAAV-EGFP; n = 9) min, *P* = .31, Figure 5F]. LH pulse amplitude was not affected in any treatment group (data not shown).

## Discussion

This is the first study to use intranuclear injections of antisense kisspeptin in juveniles to evaluate the distinct, regional roles of ARC vs AVPV kisspeptin in puberty and reproductive function. By knocking down but not completely ablating kisspeptin expression from pnd 10, some physiological function is preserved, and the potentially confounding effects of developmental compensation or redundancy are limited. Stereotactically injected rAAV-kisspeptin-AS resulted in a 37% knockdown of AVPV and 32% reduction of ARC kisspeptin content, comparable with that found using this methodology in adult Wistar rats (27).

AVPV injection of rAAV-kisspeptin-AS delayed day of vaginal opening and first estrus by an average 3.4 and 3.8 days, respectively. There was no effect of ARC knockdown on either measure, despite the close association of puberty with increasing pulse generator frequency in rats (30, 40). Neither experiment was confounded by changes in body weight, a critical factor in pubertal timing in rodents. Although both vaginal opening and first estrus are surrogate end point measurements, vaginal opening signifies the rising estrogen levels necessary for puberty initiation, and first vaginal estrus indicates complete hypothalamic-pituitary-gonadal (HPG) axis maturation (49). Given the estrogenic nature of these markers, the known role of estrogen in the maturation of AVPV kisspeptin over puberty (7, 12, 35–39), the increases in AVPV *Kiss1* expression that occur before puberty in mice (41), the lack of significant ARC kisspeptin pubertal maturation (33, 37), and the role of AVPV kisspeptin in estrogen-positive feedback upon GnRH neurons (15), it is perhaps unsurprising that it is AVPV and not ARC kisspeptin that appears to be fundamental for puberty onset. However, it is possible that the ARC knockdown stimulates a greater degree of compensation than AVPV or that AVPV-derived Kiss1 present in the ARC (39) plays a significant role or that the residual function remaining after ARC rAAV-kisspeptin-AS is sufficient for maintaining puberty onset, although the abnormal cyclicity and LH pulse data observed after the ARC rAAV-kisspeptin-AS administration suggest that the magnitude of knockdown achieved can significantly influence the HPG axis. Although the effect of ARC rAAV-kisspeptin-AS administration on pulse interval in OVX rats is relatively small, it is possible that it would result in larger effects in intact rats.

Whole-body congenital ablation of most Kiss1/Kiss1r-expressing cells failed to significantly impact puberty timing, estrous cyclicity, basal LH, or subsequent fertility in mice. However, carrying out such an ablation at pnd 20 did have a significant impact on puberty and fertility (50). This suggests that if the insult occurs early enough, functional compensatory mechanisms may restore the deficit, safeguarding reproductive function. Whole-cell ablation removes all other signaling molecules produced by that cell and may therefore trigger a greater level of compensation than other knockout methods. Multiple redundancies may further safeguard fertility. It has been shown that only 34% of GnRH is essential for LH surges and successful ovulation in mice (51). Knockdown of 95% of kisspeptin expression had no significant impact on puberty onset, estrous cyclicity, or basal LH in mice, although fecundity was reduced (52). Therefore, either only 5% of murine kisspeptin is necessary for full HPG axis function or there is compensation, for instance via NKB, glutamate or increasing GnRH neuron sensitivity to the remaining kisspeptin over time (52). These results may also simply reflect the general sensitivity of the Kiss1r to even low levels of kisspeptin (36).

In contrast, a previous *Kiss1/Kiss1r* mouse gene knockout did report delayed vaginal opening, absent estrous cycles, and infertility (53); the greater degree of gene knockout and the reported heightened GnRH response to kisspeptin could explain the discrepancy in their results and support the hypothesized role of compensation. Two studies have recently shown that kisspeptin signaling through Kiss1r on the GnRH neuron is essential for reproductive function; GnRH neuron-specific deletion of the Kiss1r resulted in absent puberty over the 6-month experimental period, no estrous cyclicity, and infertility (13). A 64% reduction in murine GnRH neuron Kiss1r resulted in delayed day of vaginal opening, abnormal estrous cyclicity, infertility, and reduced basal LH (14). This relationship is causal because the phenotype was reversed on reinstatement of the Kiss1r (13).

Puberty halts in ERα knockout mice before first estrus (54). In line with our results, an estrogen-dependent amplification mechanism may therefore exist, stimulating GnRH neurons, via ERα on AVPV kisspeptin neurons, to reach a mature state. Specific prepubertal ARC estrogen-inhibition, preventing GnRH pulse generation and gonadotropin production in the juvenile period, may be lifted by increasing activation of GnRH neurons via AVPV kisspeptin (55). The estrogen/kisspeptin-independent trigger driving this activation is unknown; glutamate, γ-aminobutyric acid (GABA), and epigenetic regulation of polycomb group genes *Eed* and *Cbx7* are currently being investigated (56, 57). Repeats of our methodology using antisense ERα, glutamate, GABA, and regulators such as neuropeptide Y *inter alia*, may provide information regarding the hierarchy of action, the role of critical windows (56), and the time line for this process.

There is a general shift from inhibition to stimulation of GnRH neurons over puberty, via changes in GABA, glutamate, and kisspeptin signaling as well as glial cell interactions (58–60). GABA and glutamate are also components of a complex interplay between KNDy neurons and other potential afferents acting upon GnRH dendrons or at the median eminence to stimulate coordinated pulsed secretions of GnRH. Where ARC kisspeptin stimulation sits and its hierarchical importance within this is not fully understood. Individually knocking down these alternative regulators using our methodology may also aid the piecing together of the control mechanisms of pulse generation.

Both normal GnRH/gonadotropin surge (32) and pulse (26) generation, supported via Kiss1-Kiss1r signaling in the AVPV and ARC, respectively, are necessary for normal estrous cyclicity. In accord with this, we found abnormal cycles in both rAAV-kisspeptin-AS groups. In both the ARC and AVPV groups, rAAV-kisspeptin-AS extended cycle length, in particular increasing the percentage of time spent in the estrous and metestrous stages, whereas time spent in diestrus was approximately halved across both experimental groups. Lapatto et al (53), Popa et al (52), and Mayer and Boehm (50) report cycles with some prolonged estrous-phase vaginal cornification, implying estrogenic stimulation has occurred (29) at a level insufficient for complete cycles of folliculogenesis and ovulation, reflected in the absent/reduced corpora lutea counts in the former two studies. *Kiss1*^−/−^/*Kiss1r*^−/−^ mice (29) and kisspeptin ERα knockout mice (54) move between the estrous and diestrous phases but remain anovulatory. GnRH knockdown mice enter puberty but are incapable of producing an LH surge, are consequently anovulatory, and fail to exhibit estrous cycles (51). Taken with our results, these studies suggest that some basal gonadotropin secretion remains in *Kiss1/Kiss1r* knockout mice, permitting incomplete rounds of folliculogenesis in which the animals get stuck in cornified preovulatory phases because the estrogen-kisspeptin-GnRH stimulus essential for ovulation is inhibited (29, 53). Our data indicate that a high level of such stimulation is required because approximately 35% of kisspeptin knockdown in either ARC or AVPV nuclei resulted in kisspeptin levels insufficient for normal cyclicity. The lack of spread of the viral vector between the injection sites in the ARC and AVPV and the lack of change in kisspeptin-IR in the opposite nucleus to where the virus was injected suggests that there is unlikely to be any significant cross-contamination between these key kisspeptin-containing nuclei.

Our AVPV data are in keeping with the loss of estrous cyclicity described after POA lesioning (61, 62) and POA kisspeptin antagonist administration (17). Similarly our ARC results correlate with the prolonged estrous cycles found after ARC lesioning (63) and the disruption in cyclicity we described after rAAV-kisspeptin-AS in adult rat ARC nuclei (27). Increased LH interpulse interval caused by ARC rAAV-kisspeptin-AS may have impaired tonic release of gonadotropins and folliculogenesis, and the inhibition of LH surges after both ARC and AVPV rAAV-kisspeptin-AS may have prevented normal ovulation.

LH surges are conventionally associated with AVPV kisspeptin and can be reliably generated using estrogen in OVX mice, which stimulates c-FOS expression in GnRH and AVPV kisspeptin neurons. This does not occur in *Kiss1* and *Kiss1r* null mice (64, 65). LH surges were measured in gonadally intact animals and so are as precise a measurement of spontaneous surge activity as possible. Only one rat in the AVPV rAAV-kisspeptin-AS group exhibited an LH surge correctly occurring on proestrus, which was of normal amplitude. In the ARC knockdown group, all rats had LH surges occurring with normal incidence on proestrus but with significantly attenuated amplitudes.

AVPV neurons have connections with ipsilateral arginine vasopressin neurons in the suprachiasmatic nucleus, implicated in circadian timing (66, 67). In rats the LH surge occurs in the afternoon of proestrus. The circadian rhythm of kisspeptin expression is largely estrogen dependent, with estrogen stimulating both transcription of and GnRH responsiveness to kisspeptin, whereas GnRH neurons have a time-specific sensitivity to arginine vasopressin and kisspeptin (68). Estrogenic stimulation, time gating, and additional circadian signaling from the suprachiasmatic nucleus coalesce to control preovulatory LH surge timing (69, 70), triggering ovulation. The lack of normally occurring LH surges induced by AVPV rAAV-kisspeptin-AS supports the role of AVPV kisspeptin in coordinating surge timing. In contrast, the normal temporal occurrence of the AVPV-intact, ARC rAAV-kisspeptin-AS LH surges suggests ARC kisspeptin has no influence over this AVPV circadian function, in line with our previous findings (27).

Because the only LH surge to occur in the AVPV group was of normal amplitude but the ARC rAAV-kisspeptin-AS LH surges were all attenuated, we may infer that ARC kisspeptin has an amplificatory role in surge generation. Rodent ARC KNDy projections extend to GnRH neurons at the median eminence (21, 71, 72) and perhaps also to an ill-defined point along the dendron projection (73). Knocking down ARC kisspeptin may inhibit this amplification and thus surge amplitude, through a more general disturbance of GnRH neuron activity and hence gonadotrophic hormone support to the ovary. Indeed, selective ARC KNDy neuron ablation markedly reduced circulating levels of LH in OVX rats (74). It is of note that in the present study, we did not observe a significant change in ARC-NKB content after knockdown of kisspeptin, thus making it unlikely that the phenotype observed reflects altered action of NKB on gonadotropin release. Although previously we did not find attenuation of the estradiol-induced LH surge using this knockdown method in adult ovariectomized rats (27), ARC kisspeptin knockdown in the juveniles did significantly reduce proestrous estradiol levels by approximately 30%. These data raise the question whether the attenuated LH surge amplitude after ARC kisspeptin knockdown could be related to the lower levels of estradiol extant at proestrus in these animals rather than simply a direct effect of ARC Kiss1 per se on the GnRH/LH surge process. It would be of interest in future work to analyze the FOS expression of ARC kisspeptin neurons during the LH surge as well as to use intra-ARC kisspeptin antagonists to see whether these LH surge findings are duplicated because a previous study has tended to focus on the AVPV when analyzing LH surges. An additional follow-up study using concomitant ARC and AVPV rAAV-kisspeptin-AS to see whether the impact upon LH surges and cyclicity is compounded would also be of interest because recent general kisspeptin knockdown studies have measured basal LH but not specifically LH surges and pulses (13, 14, 52–54).

ARC rAAV-kisspeptin-AS modestly but significantly extended the interpulse interval in the now OVX adult rats, decreasing LH pulse frequency as described previously (27). Monitoring of LH pulse frequency in the KNDy neuron ablation model of Mittelman-Smith (74) would be an intriguing follow-up study. Changes in LH pulse frequency were not found after AVPV antisense kisspeptin, in line with the classically distinct role of ARC and not AVPV kisspeptin in GnRH/LH pulse regulation across many species (75–77). The remaining ARC kisspeptin may be insufficient for maintaining adequate folliculogenesis and gonadotropin secretion, and this may underlie the reduced circulating levels of estradiol and attenuated LH surges. Kisspeptin mRNA and immunoreactivity within the ARC decrease after acute inflammation (78), during lactation (21), and under stress, and this is associated with a decreased LH pulse frequency (79), which would reduce the likelihood of pregnancy in potentially injurious conditions. A comparable ARC kisspeptin reduction is reported herein and also by Beale et al (27), which may support this theory.

Our data fit well with the developing rodent KNDy-GnRH dendron mode, which has shown that pulsed ARC KNDy neuron kisspeptin acting through Kiss1r on GnRH dendrons is crucial for GnRH pulse generation, although it is probably not the pulse generator per se (19, 73) because the electrical recording of ARC kisspeptin/KNDy neuronal activity revealed the majority to be silent or to fire irregularly (80, 81) and no kisspeptin neurons spontaneously burst fire ex vivo, thought to underlie neuropeptide release (82). KNDy neurons may instead comprise a major amplifier of GnRH dendron autonomous pulse generation.

In conclusion, we have shown that a relatively modest 37% reduction in AVPV kisspeptin delayed puberty onset and significantly impaired LH surges and estrous cycles, whereas a comparable reduction in ARC kisspeptin does not impact on pubertal timing but significantly impairs LH pulsatility, attenuates LH surge production, and impairs estrous cyclicity. These results might suggest that the role of AVPV kisspeptin in the control of pubertal timing is particularly sensitive to perturbation. Our findings support the classical division between ARC and AVPV in the control of LH pulses and surges distinctly, with the added caveat that ARC kisspeptin may have a novel amplificatory role in LH surge production. Both ARC and AVPV kisspeptins appear important for maintaining normal estrous cyclicity. As we have concluded previously (27), it seems that kisspeptin expression must be kept within a fairly narrow range to maintain normal fertility as well as achieve timely puberty.
